# Investigating attitudes, skills, and use of evidence-based practice among Norwegian chiropractors; a national cross-sectional study

**DOI:** 10.1186/s12913-023-09354-2

**Published:** 2023-04-20

**Authors:** Birgitte Lawaetz Myhrvold, Iben Axén, Matthew J Leach, Tobias Sundberg, Anne Marie Gausel

**Affiliations:** 1 Department of Interdisciplinary Health Sciences, Institute of Health and Society, University of Oslo, P.O. Box 1089, Blindern, Oslo, 0317 Norway; 2 Et Liv I Bevegelse (ELiB), The Norwegian Chiropractic Research Foundation, Oslo, Norway; 3 Unit of Intervention and Implementation Research for Worker Health, Institute of Environmental Medicine, Karolinska Institutet, Nobels väg 13, 171 77, Stockholm, Sweden; 4 National Centre for Naturopathic Medicine, Southern Cross University, Military Road, East Lismore, NSW Sydney, 2480 Australia; 5 Musculoskeletal & Sports Injury Epidemiology Center, Department of Health Promotion Science, Sophiahemmet University, Box 5605, Stockholm, 114 86 SE Sweden; 6 Department for Caring and Ethics, Faculty of Health Sciences, University of Stavanger, Stavanger, Norway; 7 Department of Obstetrics and Gynecology, Stavanger University Hospital, Stavanger, Norway

**Keywords:** Evidence-based practice, Attitudes, Skills, Use, Barriers, Facilitators, Norwegian, Chiropractors

## Abstract

**Background:**

Evidence-based practice (EBP) is essential in improving the quality of healthcare and of importance for all health care personnel. No study in Norway has investigated attitudes, skills and use related to EBP among chiropractors. The aim of this study was to describe Norwegian chiropractors’ attitudes, skills, and use of EBP, as well as the barriers and facilitators to their use of EBP.

**Methods:**

A national cross-sectional survey, the online version of the Evidence Based practice Attitudes & Utilisation SurvEy (EBASE), was sent by email to 770 Norwegian practicing chiropractors, all members of the Norwegian Chiropractic Association. Three EBASE sub-scores were generated (Attitudes, Skills and Use), and the demographic characteristics of the sample were reported. Linear regression analyses were conducted to examine the association between responses of the three sub-scores and demographic characteristics. Information on main barriers and facilitators of EBP was collected and described.

**Results:**

A total of 312 (41%) chiropractors responded to the survey, and 95% agreed that EBP is necessary for chiropractic practice. While overall use of EBP activities was low participants were interested in learning and improving their skills to incorporate EBP into practice. Chiropractors’ attitudes, skills, and use of EBP were positively associated with being female and having spent more than one hour per week on research, but negatively associated with having practiced more than 10 years. Main barriers of EBP were lack of skills to critically evaluate, interpret, and apply research findings to practice. Main facilitators of EBP included access to the internet and free online databases in the workplace.

**Conclusion:**

Although chiropractors in Norway reported positive attitudes and moderate skills in EBP, their use of EBP activities was limited. The main barriers and facilitators to EBP were primarily related to perceived skills deficits, whilst enablers of EBP were mostly related to infrastructure requirements.

## Background

The foundation of evidence-based practice (EBP) is the integration of the best available research evidence into clinical practice to improve both health outcomes and quality of care for individual patients [1, 2]. In addition to using the best available evidence from research, evidence-based healthcare considers the clinician’s clinical expertise and the patient’s background and preferences when deciding on the approach to management [1, 2]. Contemporary healthcare personnel are expected to follow evidence-informed clinical recommendations and guidelines so that clinicians and patients know that the most up-to-date and best practice treatment strategies are provided [3].

Previous surveys on EBP readiness of manual therapy professions conducted in Sweden [4], Canada [5], the United States of America [6, 7], Italy [8], Spain [9], the United Kingdom [10], and Australia [11] indicate that attitudes towards EBP uptake are generally positive, and that skills in acquiring research evidence are high, but that use of research evidence in clinical practice is rather low. Favourable attitudes toward EBP, and higher skills in, and use of EBP, have been shown to be associated with gender, age, educational level, membership of a professional association, involvement in research, publication of a paper, and/or teaching in the higher education sector or post-graduate courses [4, 5, 8, 9, 11]. The clinical environment and professional activities (such as working with conventional healthcare providers, number of hours in practice, undertaking less than 20 consultations per day, no onsite imaging, ordering less radiography and having a focus on musculoskeletal conditions) are also found to be associated with more favourable EBP attitudes, higher EBP skill-level and increased use of EBP [4, 5].

Surveys of manual therapy professions to date indicate that access to workplace internet and free online databases are frequently favoured facilitators of EBP uptake, whereas lack of time and lack of clinical evidence in the profession have been found main barriers to delivering EBP [4, 6, 8, 9, 11, 12]. While current evidence suggests that skills, attitudes, and use of EBP among manual healthcare professions is consistent, it would be inappropriate to generalise these findings to all manual healthcare professions, in all jurisdictions. Accordingly, this study seeks to understand the nuances of EBP uptake among an under-researched group - Norwegian chiropractors.

Chiropractic is an allied health profession with a scope of practise that largely focuses on musculoskeletal conditions [13]. In Norway, the profession is well integrated in the public health care system. In short, chiropractic care is partly reimbursed, and chiropractors are authorized to refer for imaging or to medical specialists, and to issue sickness-certification for musculoskeletal disorders. There is, however, no chiropractic education in Norway, and therefore only modest academic integration.

We also know little of EBP uptake within the Norwegian chiropractic profession. Only one previous survey, from 2014, has described Norwegian chiropractors attitudes towards clinical guidelines and research participation [14]. The survey found that the majority of chiropractic practitioners were familiar with Norwegian clinical guidelines for low back pain [15], and were positive towards research [14]. Nevertheless, that survey focussed primarily on research rather than EBP. In light of this knowledge gap, it is clear that a comprehensive understanding of the attitudes, skills and use of EBP among Norwegian chiropractors is needed. Knowledge of the barriers and facilitators to EBP uptake also need to be understood to develop appropriate and nuanced strategies to enhance implementation of EBP into Norwegian chiropractic practice.

### Aim and objectives

The overall aim of this study was to describe Norwegian chiropractors’ attitudes, skills, and use of evidence-based practice, as well as the barriers and facilitators to the use of EBP in the Norwegian chiropractic context. Specifically, this study aimed to address the following objectives:

- attitudes toward EBP,
- levels of perceived skill in EBP,
- levels of engagement in EBP activities,
- facilitators of, and barriers to EBP uptake, and.
- attitudes, skills, and use of EBP, and their association with demographic factors.

## Methods

### Design

An online, descriptive cross-sectional survey.

### Sample and setting

The target population consisted of 770 Norwegian practising chiropractors registered with the Norwegian Chiropractic association as of June 2021. No exclusion criteria were applied. We used the method by Riley et al. to calculate for the required sample size for multivariable linear regression modeling [16]. In the present study, seven candidate demographic variables (that included 20 parameters) were selected a priori based on previous literature. We pre-specified the anticipated R^2^ (0.8) and used mean and standard deviation of outcomes in this study sample. This specified a required sample size of 254. Our total sample size included 312 chiropractors and was within acceptable limits.

### Description of survey and variables

The Evidence-Based practice Attitude and utilization SurvEy (EBASE) [17] is an instrument designed to measure attitudes, skills and use of EBP among health care professionals. The original EBASE instrument has shown good internal consistency, construct and content validity and demonstrated acceptable test-retest reliability [17, 18]. The EBASE is divided into seven parts, including.

- Part A: attitudes (10 items, rated using a 5-point Likert scale, ranging from “Strongly disagree” to “Strongly agree”),
- Part B: skills (13 items, rated using a 5-point Likert scale, ranging from “Low” to “High”),
- Part C: education and training (5 multiple-choice items),
- Part D: use (10 items, rated based on number of articles read/reviewed, performing certain EBP-related activities in practice, information sources used to inform clinical-decision making, and estimated percentage of practice based on clinical research evidence [i.e., evidence from clinical trials]),
- Part E: barriers (13 items, rated using a 4-point Likert scale, ranging from “No barrier” to “Major barrier”).
- Part F: facilitators (10 items, rated using a 4-point Likert scale, ranging from “Not useful” to “Very useful”) and.
- Part G: demographics (14 multiple-choice items and 1 open-text item).

Items from three of the survey parts can be generated into sub-scores, as described elsewhere [19], and summarised below:

- Part A: attitude sub-score, sum of the first 8 items, ranging from 8 (predominantly strongly disagree) to 40 (predominantly strongly agree).
- Part B: skills sub-score, sum of all 13 items, ranging from 13 (primarily low-level skill) to 65 (primarily high-level skill), and.
- Part D: use sub-score, sum of the first 6 items, ranging from 0 (mainly infrequent use) to 24 (mainly frequent use).

### Translation of the questionnaire

The survey was translated from the English and Swedish versions to Norwegian by members of the research team who were fluent in these languages. After the translation, the questionnaire was pilot tested on 2 selected chiropractors. Suggestions for minor modification (e.g., improved explanation of questions, adaption to typical Norwegian) were integrated into the final version of the survey.

### Recruitment and data collection

Data collection were handled by a digital questionnaire created with Nettskjema.no, survey solution developed and hosted by the University of Oslo, Norway (nettskjema@usit.uio.no). The link to the survey was sent by email to all practicing chiropractors that were members of the Norwegian Chiropractors’ Association (n = 770) in the fall of 2021. The email contained information on the purpose of the study, survey composition, informed consent considerations, reporting of results, storage of data, and ethics. Verbal advertisement and encouragement to participate in the survey were given during the annual National Norwegian chiropractic Conference. Several participation advertisements, encouragement messages and reminders to complete the EBASE survey were distributed before and during data collection. These were sent by email or as small films and personal reminders posted on relevant social media groups (Facebook and Messenger), using collegial language (“Hi, we hope that many of you will complete the EBASE survey to understand how we use evidence in our daily practise”). Data collection took place between October and November 2021.

### Statistical analysis

Data from the online survey was imported into “Tjenester for Sensitive Data” (TSD), owned by the University of Oslo. The TSD (in Norwegian, Service for Sensitive Data) service is designed for storing and post-processing sensitive data in compliance with the Norwegian “Personal Data Act” and “Health Research Act”. Statistical analyses were performed on the TSD facilities. There were no missing data, as all items were made compulsory. Categorical data were described using frequencies and percentages. Medians and the interquartile range (IQR) were used for non-normally distributed data. Linear regression models explored associations between a priori selected demographic variables [i.e. gender (female/male), age (20–39/40–59/60 + years), years in practice (0–5/6–10/11–15/16 + years), clinical setting context (solo/with chiropractors/with conventional healthcare providers/with complementary medicine (CM)/with CM and conventional healthcare providers), country of chiropractic education (England/Denmark/USA/Australia) and hours dedicated to research per week (none/>1)], and each of the following: attitudes sub-score, skills sub-score and use sub-score. Previously, these variables have shown to be associated with attitudes, skills, and use of EBP [4, 8]. Selection of the best model was determined by variable selection after performing a multivariable linear regression using an all-subsets variable selection method. The leaps-and-bounds algorithm was used to determine the best model subsets and the highest Adjusted R^2^ was used to select/choose the best linear regression model. We set the significance level at 5% for all tests and performed all analyses in STATA/SE 16 (STATA Corp, College Stations, TX).

### Ethics

Study participation was anonymous and voluntary, and no ethical approval was needed according to Norwegian law [20]. The study was carried out in accordance with ethical guidelines [21]; and informed consent was obtained online by all participants before they could enter the survey.

## Results

### Demographic characteristics

The response rate was 41% (312/770 chiropractors). Characteristics of the participating chiropractors are shown in Table 1. Men and women were approximately equally distributed in the sample and were predominantly between the ages of 30–50 years (70%), with over one-half (55%) having practiced for more than 11 years. Most chiropractors (68%) held a bachelor’s or a higher-level graduate degree. The greatest proportion of chiropractors worked in a setting with a group of conventional healthcare providers (45%), spending 16–45 h per week in clinical practice. Most chiropractors resided in the counties of Viken (20%), Oslo (14%) or Rogaland (14%). Few chiropractors participated in research (15%) or taught in higher education (3%).

**Table 1:** Participants’ demographic characteristics (n = 312)

Characteristics	Frequency, n (%)
**Age**, n (%)	
*20–29 years*	41 (13)
*30–39 years*	129 (41)
*40–49 years*	90 (29)
*50–59 years*	36 (12)
*60 + years*	16 (5)
**Gender**, n (%)	
*Male*	174 (56)
*Female*	134 (43)
*Neutral gender*	2 (0.5)
*Do not wish to state*	2 (0.5)
**Highest qualification obtained**, n (%)	
*Vocational Degree/Diploma*	0
*University or College Certificate/Diploma*	99 (32)
*Bachelor’s degree*	6 (2)
*Master’s degree (2 years)*	197 (63)
*PhD/Doctorate*	5 (1.5)
*Other*	5 (1.5)
**Years since receiving highest qualification**, n (%)	
*0 years*	14 (5)
*1–5 years*	62 (20)
*6–10 years*	90 (29)
*11–15 years*	62 (20)
*16 + years*	84 (27)
**Years practiced in the (clinical) field of chiropractic**, n (%)	
*0 years*	11 (4.5)
*1–5 years*	46 (15)
*6–10 years*	82 (26)
*11–15 years*	71 (22.5)
*16 + years*	102 (33)
**Hours per week in clinical (chiropractic) practice**, n (%)	
*0 h*	5 (1.5)
*1–15 h*	6 (1.5)
*16–30 h*	63 (21)
*31–45 h*	207 (66)
*46 + hours*	31 (10)
**Hours per week participating in research**, n (%)	
*0 h*	263 (85)
*1 + hours*	49 (15)
**Hours per week teaching higher education**, n (%)	
*0 h*	303 (97)
*1–15 h*	9 (3)
**What type of treatments/management are included in your toolbox as alternatives you can offer in initial chiropractic consultation**, n (%)	
*Joint manipulation (e.g., HVLA)*	305 (98)
*Exercise and physical activity advice or instruction*	305 (98)
*Home exercise and ADL advice or instruction*	304 (97)
*Referral to other healthcare provider*	299 (96)
*Trigger point therapy*	292 (94)
*Joint mobilisation*	269 (87)
*Ergonomic advice or instruction*	268 (86)
*Health/lifestyle advice or instruction*	269 (86)
*Physical exercise / rehabilitation training*	261 (84)
*Massage/soft-tissue mobilization*	250 (80)
*Stretching*	246 (79)
*Traction*	245 (78)
*Referral to other health service*	224 (72)
*Non-prescription pharmaceutical advice or instruction*	188 (60)
*Dietary advice or instruction*	169 (54)
*Taping*	152 (49)
*Other treatment/management*	100 (32)
*Acupuncture*	95 (30)
*Heat/cold treatment*	88 (28)
*Nutritional supplementation advice*	84 (27)
*Laser therapy*	35 (11)
*TENS*	19 (6)
*Ultrasound*	5 (2)
**Clinical setting in which chiropractic is predominantly practiced**, n (%)	
*With a group of conventional health providers*	140 (45)
*With CM & conventional health providers*	73 (23)
*With a group of chiropractors*	62 (20)
*Solo practice*	26 (8)
*Other*	1 (0.3)
*With a group of CM providers*	10 (3)
*Within an educational institution (e.g., university)*	0
**County regions of Norway**, n (%)	
*Agder*	22 (7)
*Innlandet*	17 (5)
*Møre og Romsdal*	22 (7)
*Nordland, Troms og Finmark*	10 (3)
*Oslo*	55 (17)
*Rogaland*	43 (14)
*Vestfold og Telemark*	25 (8)
*Trøndelag*	25 (8)
*Vestland*	32 (10)
*Viken*	61 (20)
**Geographical region**, n (%)	
*City (Central business district)*	238 (76)
*Suburbs*	62 (20)
*Rural/remote region*	12 (4)
**Education land**, n (%)	
*Great Britain*	194 (62)
*Denmark*	50 (16)
*USA*	38 (12)
*Australia*	29 (9)
*Norway*	1 (0)

HVLA high-velocity low amplitude, ADL Activities of daily living, CM Complementary medicine, TENS Transcutaneous electrical nerve stimulation

### Attitudes toward EBP

Chiropractors’ attitudes toward EBP were positive with a median sub-score of 33, (IQR (30–36); range 8–40; scores ranging between 32.0 and 40.0 are indicative of predominantly agree to strongly agree) (Table 2).

**Table 2:** The median, IQR and range for the Attitudes, Skills, and Use sub-scores

	Median sub-score	IQR	Range
Variable			
Attitudes	33.0	(30–36)	(8–40)
Skills	40.5	(35–47)	(13–65)
Use	8.0	(5–15)	(0–24)

Almost all chiropractors agreed or strongly agreed that EBP was necessary in chiropractic practice (95%), EBP assists clinical decision-making (94%), and EBP improves the quality of patient care (89%), (Table 3). The majority agreed that research findings are important in practice (96%), and they were interested in learning and improving their skills to incorporate EBP into practice (95%). More than one-half (55%) disagreed or strongly disagreed that EBP placed an unreasonable demand on their practice, and 55% of chiropractors disagreed that there is a lack of evidence from clinical trials to support most of the treatments used in practice. However, chiropractors’ attitudes were divided on whether EBP considers a patient’s preference for treatment with approximately one-third disagreeing, one-third being neutral and one-third agreeing.

**Table 3:** **Respondent attitudes** (Part A) toward evidence-based practice (n = 312)

	1 Strongly Disagree n (%)	2 Disagree n (%)	3 Neutral n (%)	4 Agree n (%)	5 Strongly Agree n (%)	Median (IQR)
EBP is necessary in the practice of chiropractic	3 (1)	7 (2)	6 (2)	143 (46)	**153 (49)**	4 (4–5)
EBP improves the quality of my patient’s care	-	11 (4)	25 (8)	**143 (46)**	133 (43)	4 (4–5)
EBP assists me in making decisions about patient care	-	6 (2)	14 (4)	**152 (49)**	140 (45)	4 (4–5)
I am interested in learning or improving the skills necessary to incorporate EBP into my practice	1 (0.3)	2 (1)	14 (4)	124 (40)	**171 (55)**	5 (4–5)
Professional literature (i.e., journals & textbooks) and research findings are useful in my day-to-day practice	1 (0.3)	-	10 (3)	145 (46)	**153 (50)**	4.5 (4–5)
Prioritizing EBP within chiropractic practice is fundamental to the advancement of the profession	3 (1)	13 (4)	24 (8)	**150 (48)**	122 (39)	4 (4–5)
EBP takes into account my clinical experience when making clinical decisions	2 (1)	32 (10)	50 (16)	**138 (44)**	90 (29)	4 (3–5)
EBP takes into account a patient’s preference for treatment	4 (1)	91 (29)	**98 (31)**	73 (23)	46 (15)	3 (2–4)
There is a lack of evidence from clinical trials to support most of the treatments I use in my practice	24 (8)	**146 (47)**	57 (18)	78 (25)	7 (2)	2 (2–4)
The adoption of EBP places an unreasonable demand on my practice	61 (20)	**174 (56)**	44 (14)	29 (9)	4 (1)	4 (4–4)

*EBP* Evidence-based practice, *IQR* Interquartile range; main response in bold

Multivariable analysis between demographic characteristics and the attitude sub-score found the best subset model included age, number of years in practice, undertaking research activities, clinical setting, and country of education. The model found that spending one or more hours per week on research were significantly associated with a higher attitude sub-score (i.e. more positive attitude towards EBP) whereas having practiced between 11 and 15 years, and being educated in the USA was significantly associated with a lower attitude sub-score (Table 4).

**Table 4:** Linear regression analysis between demographic variables and Attitudes sub-score

Attitudes sub-score			
	**β**	**95% C.I.**	***P*** **-value**
Demographic variables			
**Age**			
*20–39 years*	Ref		
*40–59 years*	-0.03	-0.23, 0.17	0.774
*60 + years*	0.26	-0.07, 0.58	0.119
**Years in practice**			
*0–5 years*	Ref		
*6–10 years*	-0.10	-0.26, 0.06	0.239
*11–15 years*	-0.21	-0.40, -0.02	0.028*
*16 + years*	-0.11	-0.36, 0.14	0.400
**Research hours spent per week** (ref: none)	0.21	0.07, 0.35	0.004*
**Clinical setting**			
*Solo*	Ref		
*w/chiropractors*	0.10	-0.12, 0.31	0.385
*w/conventional health providers*	0.14	-0.06, 0.33	0.173
*w/CM*	-0.10	-0.43, 0.24	0.581
*w/CM and conventional health providers*	0.05	-0.16, 0.26	0.611
**Education**			
*England*	Ref		
*Denmark*	0.00	-0.15, 0.16	0.970
*USA*	-0.26	-0.44, -0.07	0.007*
*Australia*	0.10	-0.09, 0.29	0.324

*Statistically significant, p-value < 0.05

### Skills of EBP

The median skill sub-score (40.5 (IQR 35–47); range 13–65) was indicative of a predominantly moderate to moderate-high skill level of perceived skills in EBP (as defined by scores ranging between 39.1 and 51.9) (Table 2). Chiropractors predominantly reported a Moderate to Moderate-high level of skills across 11 of 13 areas related to EBP. The highest levels of skills in EBP (Moderate-high to High) were identifying clinical problems (71%) and knowledge gaps in practice (55%), locating professional literature (46%), online database searching (46%) and using findings from clinical research (46%). The lowest level of skills (Low to Low-moderate) was reported for the conduct of clinical research (71%) and systematic reviews (63%) (Table 5).

**Table 5:** Respondent self-reported skills (Part B) in evidence-based practice (n = 312)

	1 Low n (%)	2 Low-moderate n (%)	3 Moderate n (%)	4 Moderate-high n (%)	5 High n (%)	Median (IQR)
Identifying precise clinical questions	2 (1)	9 (3)	78 (25)	**172 (55)**	51 (16)	4 (3–4)
Identifying knowledge gaps in practice	2 (1)	12 (4)	127 (41)	**141 (45)**	30 (10)	4 (3–4)
Locating professional literature	10 (3)	45 (14)	**111 (35)**	98 (31)	48 (15)	3 (3–4)
Online database searching	14 (4)	52 (17)	**102 (33)**	85 (27)	59 (19)	3 (3–4)
Retrieving evidence	14 (4)	48 (15)	**126 (40)**	85 (27)	39 (13)	3 (3–4)
Critical appraisal of evidence	13 (4)	69 (22)	**117 (38)**	83 (27)	30 (10)	3 (2–4)
Synthesis of research evidence	8 (3)	48 (15)	**137 (44)**	91 (29)	28 (9)	3 (3–4)
Applying research evidence to patient cases	7 (2)	43 (14)	**136 (44)**	96 (31)	30 (10)	3 (3–4)
Sharing evidence with colleagues	20 (6)	76 (24)	**112 (36)**	80 (26)	24 (8)	3 (2–4)
Using findings from systematic reviews	21 (7)	42 (13)	**123 (39)**	102 (33)	24 (8)	3 (3–4)
Using findings from clinical research	9 (3)	32 (10)	**128 (41)**	115 (37)	28 (9)	3 (3–4)
Conducting clinical research	**130 (42)**	89 (29)	58 (19)	26 (8)	9 (3)	2 (1–3)
Conducting systematic reviews	**102 (33)**	94 (30)	76 (24)	32 (10)	8 (3)	2 (1–3)

IQR Interquartile range; main response in bold

Multivariable analysis between demographic characteristics and the skill sub-score found the best subset model included gender, number of years in practice, country of education and undertaking research activities. The model found that being female was significantly associated with a higher skill sub-score (i.e., higher level of perceived skills in EBP) whereas spending one or more hours per week on research was significantly associated with a lower skill sub-score (Table 6).

**Table 6:** Linear regression analysis between Demographic variables and Skill sub-score

Skills sub-score					
	**β**	**95% C.I.**	**P-value**		
Demographic variables					
**Gender** (ref: female)	0.21	0.10, 0.32	0.000*		
**Years in practice**					
*0–5 years*	Ref				
*6–10 years*	-0.07	-0.24, 0.10	0.415		
*11–15 years*	-0.14	-0.31, 0.04	0.131		
*16 + years*	-0.08	-0.24, 0.09	0.361		
**Research hours spent per week** (ref: none)	-0.35	-0.50, -0.20	0.000*		
**Clinical setting**					
*Solo*	Ref				
*w/chiropractors*	0.00	-0.22, 0.22	0.993		
*w/conventional health providers*	0.04	-0.16, 0.25	0.669		
*w/CM*	-0.01	-0.36, 0.34	0.957		
*w/CM and conventional health providers*	-0.04	-0.26, 0.17	0.699		
**Education**					
*England*	Ref				
*Denmark*	-0.05	-0.21, 0.10	0.496		
*USA*	-0.09	-0.27, 0.09	0.320		
*Australia*	-0-19	-0.38, 0.01	0.064		

*Statistically significant, p-value < 0.05

### Use of EBP

Chiropractors’ median use sub-score of 8 (IQR 5-14.5; range 0–24) reflected a moderate-low level of EBP activities in the previous month. A score between 6.1 and 12.0 is suggestive of a moderate-low level of use) (Table 2). Engagement in EBP activities was variable, but most chiropractors (50–65%) reported that they had engaged in EBP activities 1 to 10 times over the past month. Specifically, the activities most engaged were using online search engines to search for practice-related literature, consulting a colleague or industry expert to assist clinical decision-making, and using professional literature or research findings to assist clinical decision-making. Most chiropractors (75%) also indicated that a moderate to large proportion of their practice was based on clinical research evidence. Between 5 and 28% of chiropractors reported they had not engaged in any of the EBP activities in the previous month (Table 7).

**Table 7:** **Respondent use** (Part D) of evidence-based practice (i.e. number of times each activity was undertaken over the last month) (n = 312)

	0 0 times n (%)	1 1–5 times n (%)	2 6–10 times n (%)	3 11–15 times n (%)	4 16 + times n (%)	Median (IQR)
I have used an online search engine to search for practice related literature or research	17 (5)	**115 (37)**	55 (18)	29 (9)	96 (31)	2 (1–4)
I have used professional literature or research findings to assist my clinical decision-making	41 (13)	**133 (43)**	45 (14)	13 (4)	80 (26)	1 (1–4)
I have read/reviewed clinical research findings related to my practice	46 (15)	**152 (49)**	43 (14)	13 (4)	58 (19)	1 (1–2)
I have read/reviewed professional literature (i.e., professional journals & textbooks) related to my practice	54 (17)	**153 (49)**	40 (13)	15 (5)	50 (16)	1 (1–2)
I have consulted a colleague or industry expert to assist my clinical decision-making	19 (6)	**136 (44)**	51 (16)	18 (6)	88 (28)	1 (0–2)
I have used professional literature or research findings to change my clinical practice	52 (17)	**173 (55)**	30 (10)	21 (7)	36 (12)	1 (1–2)
I have used an online database to search for practice related literature or research	72 (23)	**119 (38)**	43 (14)	13 (4)	65 (21)	1 (1-2.5)
I have referred to magazines, layperson / self-help books, or non-government/non-education institution websites to assist my clinical decision-making	87 (28)	**121 (39)**	34 (11)	22 (7)	48 (15)	2 (1–4)

IQR Interquartile range; main response in bold

Multivariable analysis between demographic characteristics and the use sub-score found the best subset model included gender, age and number of years in practice, educational institution and undertaking research activities. The model found that being female, older, and spending one or more hours per week on research were significantly associated with a higher use sub-score (i.e. greater use of EBP in the previous month) whereas having practiced more than 6–10 years was significantly associated with a lower use sub-score (Table 8).

**Table 8:** Linear regression analyses between Demographic variables and Use sub-score

Use sub-score					
	**β**	**95% C.I.**	***P*** **-value**		
Demographic variables					
**Gender** (ref: female)	0.16	0.06, 0.27	0.003*		
**Age**					
*20–39 years*	Ref				
*40–59 years*	0.12	-0.08, 0.32	0.226		
*60 + years*	0.48	0.15, 0.81	0.004*		
**Years in practice**					
*0–5 years*	Ref				
*6–10 years*	-0.08	-0.24, 0.08	0.347		
*11–15 years*	-0.33	-0.52, -0.13	0.001*		
*16 + years*	-0.50	-0.75, -0.25	0.000*		
**Research hours spent per week** (ref: none)	0.17	0.03, 0.32	0.020*		
**Education**					
*England*	Ref				
*Denmark*	-0.11	-0.26, 0.04	0.162		
*USA*	-0.14	-0.32, 0.05	0.141		
*Australia*	0.12	-0.07, 0.31	0.213		

*Statistically significant, p-value < 0.05

Information sources that were ‘Used a lot’ or ‘Always’ by chiropractors to inform their clinical decision-making included clinical practice guidelines (62%), however, chiropractors also favoured traditional knowledge (56%), and fellow practitioners or experts (51%). Information sources ‘Never used’ or ‘Used a little’ were experimental/laboratory research (87%), trial and error (44%), and textbooks (36%) (Table 9).

**Table 9:** Information sources (Part D, separate question) used by respondents to inform their clinical decision-making (n = 312)

	Never used n (%)	Used a little n (%)	Used to a moderate extent n (%)	Used a lot n (%)	Always used n (%)
Published clinical evidence	5 (2)	51 (16)	**143 (46)**	102 (33)	9 (3)
Traditional knowledge	5 (2)	31 (10)	101 (32)	**150 (48)**	25 (8)
Clinical practice guidelines	1 (0.3)	25 (8)	92 (30)	**169 (54)**	24 (8)
Personal preference	7 (2)	33 (11)	**155 (50)**	106 (34)	10 (3)
Patient preference	2 (1)	40 (13)	**129 (42)**	118 (38)	20 (6)
Personal intuition	6 (2)	55 (18)	**141 (45)**	94 (30)	14 (5)
Fellow practitioners or experts	1 (0.3)	24 (8)	128 (41)	**147 (48)**	9 (3)
Textbooks	10 (3)	103 (33)	**135 (43)**	61 (20)	3 (1)
Trial and error	5 (2)	**130 (42)**	127 (41)	48 (15)	2 (1)
Experimental/laboratory evidence	135 (43)	**138 (44)**	29 (9)	9 (3)	0

Main response in bold

### Training and education

Of the included chiropractors, about one-half indicated that the following topics were major parts of their chiropractic education: coursework about EBP (55%), applying research evidence to clinical practice (55%), and critical thinking/analysis (56%). Only 14% of chiropractors indicated they never had any critical thinking/analysis included in their chiropractic education. Approximately one-third had never received any education on conducting systematic reviews (31%) or clinical research (27%).

### Barriers and facilitators of EBP use

The majority (55–96%) of chiropractors found that 12 of 13 potential barriers were either ‘not a barrier’ or ‘only a minor barrier’. Those factors considered by most chiropractors ***not*** to be a barrier to EBP uptake were lack of collegial support for EBP (69%), lack of profession support (57%), lack of interest for EBP (55%), lack of resources (53%), lack of relevance to chiropractic (49%), and patient preference for a specific treatment (36%). Factors largely reported as a major or moderate barrier to EBP uptake are reported in Table 10.

**Table 10:** The most frequently reported barriers and facilitators to EBP use among Norwegian Chiropractors, and the percentage of respondents who agreed

Barriers of EBP use	Facilitators of EBP use
Insufficient skills to critically appraise the literature (75%)	Access to the internet in the workplace (89%)
Lack of clinical evidence in chiropractic (74%)	Access to free online databases in the workplace (74%)
Insufficient skills to apply research findings to practice (71%)	The ability to download full-text journal articles (65%)
Insufficient skills for interpreting research (69%)	Access to online education materials related to EBP (62%)
Insufficient skills for locating research (66%)	Access to critical reviews of research evidence relevant to chiropractic (57%)
Lack of incentive to participate in EBP (66%)	Access to critical appraised topics relevant to chiropractic (53%)
Lack of time (61%)	

Most chiropractors (53–89%) found that most of the listed factors were ‘very useful’ facilitators of EBP uptake, as reported in Table 10. Conversely, factors favoured the least were access to online tools to assist with conducting critical appraisals of multiple papers related to one subject, and access to research rating tools that facilitate critical appraisal of single research papers, with only 28% and 30% reporting this as ‘very useful’, respectively.

## Discussion

This is the first study to examine Norwegian chiropractors’ attitudes, skills and use relative to EBP. The study found that Norwegian chiropractors generally report positive attitudes towards EBP and moderate to high levels of perceived EBP skills. Despite this, chiropractor participation in EBP activities over the previous month was mostly low and infrequent.

The results from this survey indicate that participating Norwegian chiropractors were relatively young and graduated recently from a European educational institution. Most worked in private practices together with other healthcare providers, with few working in solo practices. The demographic and work characteristics of the study sample were similar to those reported in the previous national survey of Norwegian chiropractors [14]. This provides support for the generalizability of our results.

### Attitudes

The positive attitudes toward EBP among participating chiropractors agree with previous studies of Swedish, Australian, American, and Canadian chiropractors [4–6, 12], as well as other health care professions [8–11]. Specifically, Norwegian chiropractors strongly believed in learning and improving their skills to incorporate EBP into practice, and strongly agreed that EBP was necessary for chiropractic practice. While few chiropractors believed that the adoption of EBP placed an unreasonable demand on their practice, almost two-thirds had indicated that lack of time was a major or moderate barrier to EBP uptake. While the reason for this inconsistent finding is unclear, it is possible that some chiropractors may have conceptualized ‘demand’ as something other than time, such as the burden of EBP on administration or management of care [22].

Norwegian chiropractor attitudes toward EBP were found to be significantly associated with engagement in research activities. These findings concur with those reported among chiropractors in Sweden and Canada [4, 5, 9, 11], as well as Italian, Spanish and Australian osteopaths [8]. This is understandable given that research training/experience facilitates the development of critical thinking skills, and enhances awareness of the interrelatedness of research and practice – both of which are necessary to implementing EBP [23].

### Skills

Participating chiropractors reported that their poorest skills in EBP related to conducting clinical research and/or systematic reviews. This is not surprising as such skills are unlikely to be developed in most chiropractic programs. By contrast, most chiropractors were comfortable with identifying clinical problems and knowledge gaps in practice, and generally judged the level of these skills as relatively high. This was also found in studies of Swedish and US chiropractors [4, 6], and may be expected as chiropractors work in a clinical environment, where these skills are frequently applied.

Overall, few chiropractors participated in research activities, and those who did, only engaged in such activities between 1 and 5 h per week. Given that most participating chiropractors worked in private practice, and not in an academic or research institution, this is to be expected as practicing chiropractors may not have the time and/or resources to engage in academic activities. An interesting finding of this study was the statistically significant inverse association between participation in research activities and perceived skill level regarding EBP. While this finding may appear illogical, it can be explained by the Dunning-Kruger effect, which states that people with high levels of skill (such as EBP-related skills) tend to underestimate their skill level, and vice versa [24]. This could also mean that participants reporting high levels of skill in EBP, may have been overestimating their skill level. This possibility should be taken into consideration when interpreting the findings of this study.

### Use

Most chiropractors indicated that a moderate to large proportion of their practice was based on clinical research evidence. This is consistent with findings from a study of Swedish chiropractors [4], yet is much higher than that reported among US and Canadian chiropractors [5, 6]. In both the US and Canadian studies, the chiropractors were relatively older and had more years in practice than our sample. This is an important point of difference, as our analysis revealed that years in practice was negatively associated with EBP use; probably because more recently completed chiropractic education programmes are more likely to incorporate content on evidence-based practise [25, 26].On the other hand, our analysis indicated that being older was significantly associated with higher use of EBP, which does appear somewhat contradictory. Although there is no clear explanation for this paradox, it is possible that the small number of chiropractors in our sample aged 60 years or above, could have been behaviourally different than the population of chiropractors in this age group, and thus, may not be representative of chiropractors aged 60 years or older. As such, this finding should be interpreted with caution.

When participating chiropractors were specifically asked about the information sources used to support clinical decision-making, the majority reported using clinical practice guidelines, followed by traditional knowledge and fellow practitioners or experts. Similar findings of using clinical guidelines, traditional knowledge and fellow practitioners to inform their clinical decision-making were reported in both the Swedish and the Australian study, however, in these studies the overall use was lower compared to our results [4, 12]. It may be a cause of concern that expert opinion and traditional knowledge are used equally to clinical guidelines and further research is needed to understand the potential implications of this to chiropractic practice in Norway.

### Barriers and facilitators

Understanding barriers and facilitators of EBP may help facilitate EBP uptake and implementation in Norwegian chiropractic practice. In our study, the major facilitators of EBP use were accessibility to the internet and online databases in the workplace. These facilitators of EBP uptake are similar across countries and manual therapy professions [4–6, 9–11], with internet access being a leading enabler across studies. Like other studies of chiropractors [4–6], barriers to EBP uptake among Norwegian chiropractors included insufficient skills to critically appraise, interpret, and apply research findings to practice, lack of clinical evidence, lack of incentive to participate in EBP, and lack of time. Fortunately, none of these barriers are unsurmountable, and may be overcome by investing in the provision of adequate evidence resources, skills development, and training [27]; in addition to providing encouragement and assistance from the profession, researchers, and knowledge hubs.

Further, approaches to overcoming these barriers should be integrated into chiropractic educational programs, as well as continuing education for practicing chiropractors. Appropriate investment in such approaches will be critical to the successful implementation of EBP in Norwegian chiropractic practice.

### Limitations and strengths

Although our study was novel, and we had employed several participation reminder strategies, the response rate was modest. Still, our response rate was similar to a previous survey of Norwegian chiropractors [14], and substantially higher than previous studies examining EBP among chiropractors in Sweden and Australia [4, 12]. Nevertheless, the sample included only 41% of the entire Norwegian chiropractor population, which may limit the generalisability of findings. On the other hand, the demographics of our sample was found to be representative of the source population in relation to age and gender [28]. Our large sample size was also a strength as this allowed us to perform linear regression analysis to better understand the association between demographic factors and attitudes, skills, and use of EBP.

The survey was based on both the Swedish and English versions of EBASE [4, 17], and although the English version has been psychometrically tested and found to be acceptable [18], the Norwegian version of EBASE has yet to be psychometrically tested. However, the Norwegian translation of the survey was undertaken by a team of experienced researchers, and pilot tested with practicing chiropractors, to mitigate the risk of translation error. Further, apart from the language, and amendments to the response options of some demographic questions (to ensure these options applied to the geographical and professional context of the study), all other items and response options in the survey (and the order of such) remained unchanged.

Due to differences in the year of publication, response rates, and context (i.e., different professional samples, differences in educational curricula, and practice type (authorized versus complementary healthcare personnel)), drawing comparisons between our sample, and those of other EBASE studies, was challenging. For instance, if a study was done some time ago, EBP might not have penetrated clinical practice to the level observed today. Another difference is the context in which the chiropractic profession practices (i.e., practicing in the national public health care system, or in private practice), as the former typically will encourage EBP use. For instance, being in solo practice was less common in Norway relative to the other studies [4, 6, 7, 12]. In fact, between 2011 and 2021, the proportion of Norwegian chiropractors in solo practice reduced from 25 to 8% [14]. In other words, the differences observed between our study and other EBASE studies may be partly due to the higher percentage of respondents in our study working in interdisciplinary environments. Moreover, the authorization of Norwegian chiropractors to refer for imaging or to medical specialists, and to issue sickness-certification for musculoskeletal disorders may contribute to differences between our study and other EBASE studies. Thus, we speculate that one is likely to discuss EBP with other health care professionals as part of everyday practise. A qualitative study suggested that the preferred qualities in chiropractors working in a multidisciplinary team included clinical expertise, safe practice, and knowledge about evidence-based treatments [29].

Concerning educational curricula, chiropractic programs are different across and even within countries, schools, and universities [26, 30]. These variations are likely to contribute to jurisdictional differences in practitioner attitude, skill, and use of EBP. In addition, some countries have governmental mandates on continued education requirements for health care professions, which may influence the extent to which practitioners engage with contemporary evidence.

### Implications

Norwegian chiropractors reported positive attitudes toward EBP, and perceived they had a good level of EBP-related skill, yet rarely used EBP. A key enabler of EBP uptake was access to the internet and open-access papers. However, the results also indicated that there was a need for relevant training to enable chiropractors to engage in EBP. Training courses that facilitate EBP skills development and use (such as workshops, journal clubs, and roadshows) have been shown to be a feasible strategy for healthcare professionals [31]. In addition, accessibility to EBP resources, such as free databases of systematic reviews, guidelines and/or randomized trials, may also support the application of EBP. However, there is no ‘one-size-fits all’ approach to EBP implementation, and most studies conclude that strategies for knowledge translation should be multi-faceted and are more effective when local facilitators and barriers are identified [31, 32]. Our study identified some barriers to EBP uptake among Norwegian chiropractors, and thus has made the first step to improving EBP implementation in this population.

Chiropractors’ adoption of EBP should be systematically included within program curricula and be prioritized and provided in a manageable form as continuing education for practicing chiropractors. Currently, there is no chiropractic education in Norway, and thus, there is no academic base for the profession to grow. However, EBP training and access to research findings are part of academicization of a profession. This may be achieved by delivering chiropractic education within a university environment. Until this is achieved, a temporary solution may be to establish a centre for professional excellence to foster the provision of continued professional development related to EBP. Such a centre could also assist in overcoming other barriers identified in this study, such as EBP incentivisation and evidence synthesis.

## Conclusions

The study provides insight into the attitudes, skills, and use of EBP among Norwegian chiropractors. Chiropractors had positive attitudes and moderate to high perceived EBP skills and believed in the value of EBP to improve outcomes and healthcare quality of individual patients. However, chiropractors had limited time and incentive to find research evidence; accordingly, use of EBP was low. The barriers to EBP were primarily related to perceived skills deficits, whilst facilitators of EBP were mostly related to infrastructure requirements.

## Data Availability

The data that supports the findings of the current study are not publicly available due to data protection policies. Data are however available from the corresponding author on reasonable request.

## Abbreviations

EBP: Evidence-based practice
EBASE: The Evidence-Based practice Attitude and utilization SurvEy
CM: Complementary medicine
HVLA: high-velocity low amplitude,
ADL: Activities of daily living,
TENS: Transcutaneous electrical nerve stimulation
SD: Standard deviation
IQR: Interquartile range
95% CI: 95% confidence interval
